# Comparison of rabbit corneal changes during different preservation techniques using optisol-GS and airlift

**DOI:** 10.1038/s41598-023-34039-5

**Published:** 2023-04-28

**Authors:** Diya Tang, Masafumi Uematsu, Kohei Harada, Yasser Helmy Mohamed, Mao Kusano, Daisuke Inoue, Takashi Kitaoka

**Affiliations:** Department of Ophthalmology and Visual Sciences, Graduate School of Biomedical Sciences, Nagasaki University, Nagasaki, 852-8102 Japan

**Keywords:** Medical research, Preclinical research

## Abstract

A previous study suggested that the airlift condition is superior to the Optisol-GS condition for preserving the limbal tissue of the human cornea. The purpose of this research is to investigate a new preservation device that preserves the cornea while separating epithelial and endothelial areas. The differences after preserving the corneal epithelium under different conditions were compared. A total of 24 corneas of New Zealand rabbits were divided into four groups in which the corneal epithelia were submersed in Optisol-GS or under airlift conditions for 1 and 2 weeks at 4 $^{\circ }$C. Transparency, optical coherence tomography (OCT), hematoxylin and eosin (H &E) staining, and epithelial migration tests were used to assess corneal status. The epithelial migration examination showed significantly greater migration ability after the airlift condition. Corneas in the 1-week Optisol-GS group were the most transparent, followed by the 1-week airlift group. OCT showed a progressive increase in corneal thickness to the end of the study. H &E staining showed that the epithelial cells retained intact cellular structure and morphology of the cells for both 1-week-preserved groups. However, there was disruption of the corneal epithelial cell structure for both 2-week-preserved groups. Corneal epithelium preserved under the hypothermic airlift condition was comparable to that under the Optisol-GS condition.

## Introduction

Corneal transplantation is often used to restore vision in patients with corneal damage, such as keratoconus, Fuchs’ dystrophy, and corneal scarring and swelling^1^.Currently, there are various types of corneal preservation methods, including short-term (storage in a moist chamber), medium-term (hypothermia), long-term (organ cultivation), and unlimited (cryopreservation)^2^. Donor corneas stored at 4 $^{\circ }$C in some commercial preparations, such as K-Sol and Optisol-GS, remain thin and clear^3^. These solutions contain osmotic agents that help limit corneal tissue swelling and offer an extended preservation time of a week to ten days^4^. In a previous study, researchers suggested that limbal tissues preserved under hypothermic airlift conditions were able to retain intact structure, normal phenotype, high viability, and the stem cell pool of limbal epithelia. However, these authors also suggested that the preservation medium, Optisol-GS, could potentially damage the cornea^5^. The goal of the current study was to develop a new corneal preservation method, and then compare corneas preserved under the new method versus those preserved using the traditional method. In addition, a new corneal preservation method that separately preserved the endothelium and epithelium of the cornea was tested. Moreover, four tests designed to compare the status of the corneas under these different storage conditions were performed.

## Materials and methods

### Preservation with artificial chamber

The artificial anterior chamber (Single-Use Artificial Chamber, Moria, Antony, France) is a device that is used to mount donor cornea and then maintain an adequate pressure for lamellar dissection or full thickness trephination. In this study, the device was modified for use in separated layer preservations.

**Figure 1:**
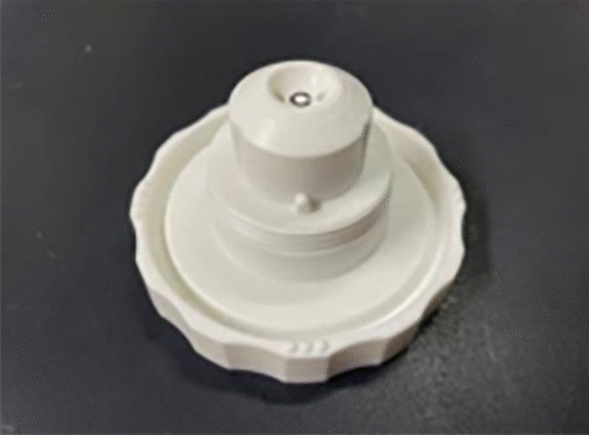
The artificial anterior chamber.

The protrusion on the top of the artificial chamber was smoothed using sandpaper to avoid mechanical damage to the cornea. One Landolt broken ring, which represented 0.15 decimal near visual acuity, was glued to the bottom of each artificial chamber for the purpose of checking the degree of button clarity (Fig. 1). All artificial chambers were disinfected with 70$\%$ alcohol and placed in a UV-irradiated room for 1 day.

### Experimental animals

Twelve male white New Zealand rabbits were individually housed in cages under controlled temperature (21 $^{\circ }$C) and humidity (50 ± 5$\%$) and a 12:12 h light/dark cycle at the Laboratory Animal Center for Biomedical Research, Nagasaki University School of Medicine. The study was started once the rabbits had reached weights of 2.0 kg, since this was the point where the corneal diameters were of suitable size for experimentation. Each rabbit had a corneal diameter equal to approximately 13 mm. All animal experiments were performed according to the Guidelines for Animal Experiments of Nagasaki University and the ARVO Statement for the Use of Animals in Ophthalmic and Vision Research, and the protocol was approved by the Regulations of Animal Care and Use Committee of Nagasaki University. This study is reported in accordance with the ARRIVE guidelines.

### Corneal preparation and preservation

**Figure 2:**
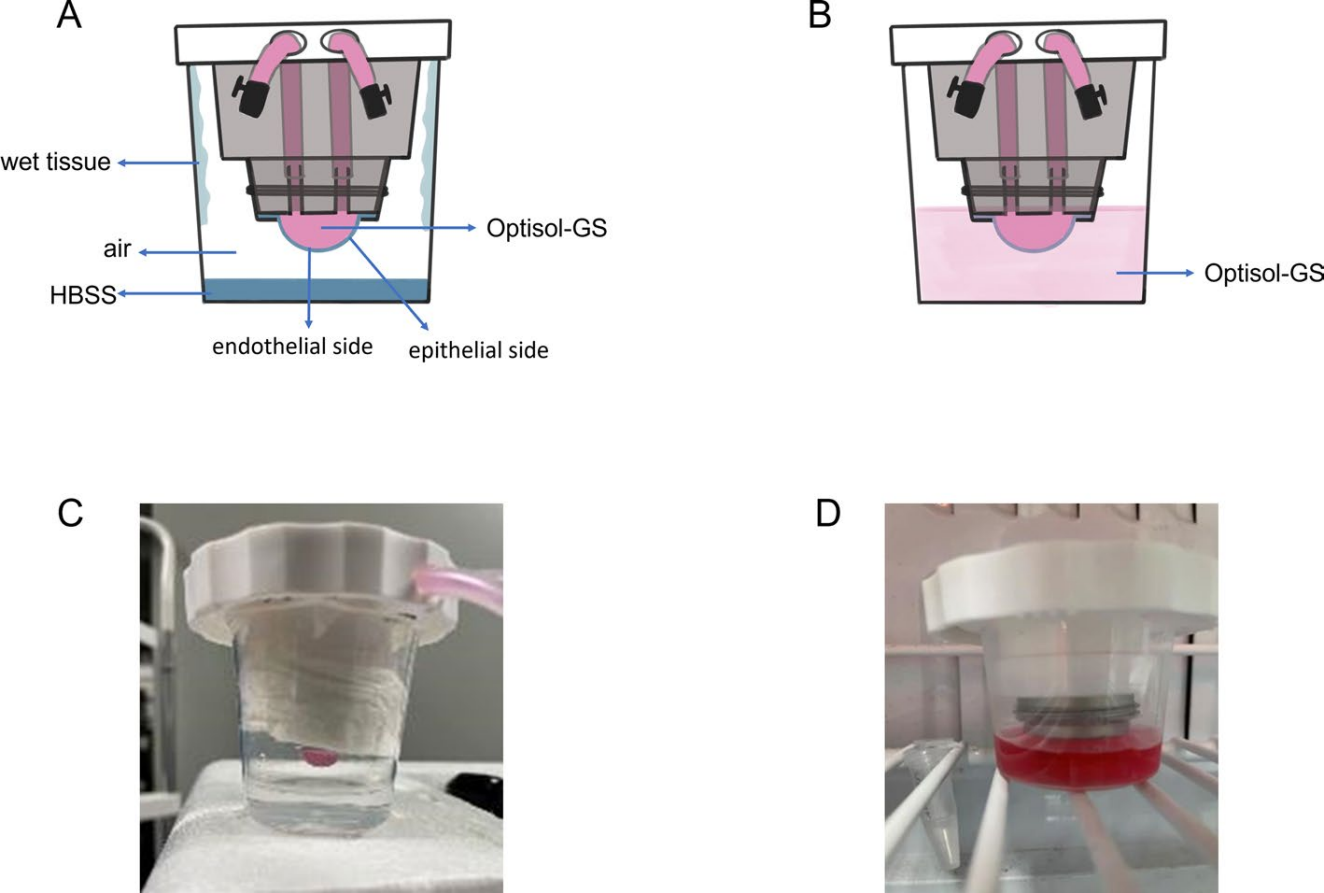
A new preservation device that preserves the cornea while separating epithelial and endothelial areas. (**A**) Schematic for the airlift group. (**B**) Schematic for the Optisol-GS submerged group. (**C**) Picture of the airlift group. (**D**) Picture of the Optisol-GS submerged group. In the Optisol-GS submerged group, both the epithelial and endothelial sides were completely submerged in the Optisol-GS. In the airlift group, the corneal endothelium was immersed in Optisol-GS, while the epithelium was exposed to the air. The humidity of the air was maintained by placing 5 mL of HBSS on the bottom of the plastic cup, with a wet paper towel placed around the inner wall of the cup.

Rabbits were anesthetized with an intramuscular injection of 30 mg/kg ketamine (Ketalar; Sankyo, Tokyo, Japan) and 5 mg/kg xylazine (Celactal; Bayer Health Care, Osaka, Japan). After being put under anesthesia, the rabbits were sacrificed with carbon dioxide. Before enucleation of the globe, 50 $\upmu$L of fluorescein solution were dropped on the surface of the cornea to check for any epithelial damage using a slit lamp. Once it was confirmed that there was no damage to the cornea, the eyeball was enucleated. The corneoscleral button with a 4-mm scleral rim was carefully excised and rinsed with Optisol-GS on a clean bench. The tissue was mounted on the artificial anterior chamber and then fixed tightly using a metal ring with a screw to avoid leakage of the Optisol-GS from the chamber. The chamber was connected to a vertically fixed infusion tube filled with Optisol-GS up to a height of 20 cm, which maintained the pressure at 15 mmHg. The artificial anterior chamber with the button was then placed upside down in a plastic cup (Fig. 2).

### Preservation groups

Twenty-four buttons were randomly divided into four groups based on the epithelial preservation method and the preservation period. The groups included: 1-week preservation Optisol-GS submerged group (1w O/O); 1-week preservation airlift group (1w A/O); 2-week preservation Optisol-GS submerged group (2w O/O); and 2-week preservation airlift group (2w A/O). All buttons were kept in a refrigerator at 4 $^{\circ }$C. In the Optisol-GS submerged groups, both the epithelial and endothelial sides were completely submerged in Optisol-GS. In the airlift groups, the corneal endothelium was immersed in Optisol-GS while the epithelium was exposed to the air. The humidity of the air was maintained by placing HBSS on the bottom of the plastic cup, with a wet paper towel placed around the inner wall of the cup. The humidity of the air was 98$\%$, as measured by a hygrometer in the preliminary experiment.

### Corneal examinations

All groups were compared with regard to transparency and corneal thickness before and after storage. Epithelial migration and hematoxylin & eosin (H &E) staining were examined after storage.

#### Transparency

**Figure 3:**
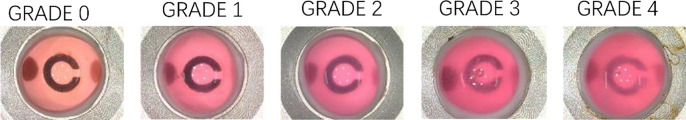
The transparency of the cornea is divided into four grades: Grade 1, almost clear; Grade 2, blurring can be found at the edge of the cornea; Grade 3, more than 50% of the cornea appears significantly blurred, but the central part retains some transparency; and Grade 4, totally blurred. Grade 0, taken at the beginning of preservation, is used as the blank control group.

Photographs of the corneas fixed in the artificial chamber were taken with a microscope that was placed in the same position and at the same magnification at the beginning and at the end of the preservation. Transparency of the cornea was divided into four grades: Grade 1, almost clear; Grade 2, blurring can be found at the edge of the cornea; Grade 3, more than 50$\%$ of the cornea appears significantly blurred, but the central part retains some transparency; and Grade 4, totally blurred. The picture of Grade 0 was used as the blank control group, which was taken at the beginning of preservation (Fig. 3). The grade number was counted as the score. Two authors, who were double-blinded, scored each picture, and if the scores were different, a third double-blinded author determined the score.

#### Corneal thickness

All corneas were examined for thickness in the central part before and after preservation using a handheld optical coherence tomography device (iVue-100, Optovue, Orlando, FL, USA).

#### H &E staining

After finishing storage, corneas were removed from the artificial chamber. One-third of the cornea was cut off and used for the sample, using Tissue-Tek O.C.T. Compound as the embedding medium. This ensured that the corneal sample would be at the optimal cutting temperature. Subsequently, the corneal samples were placed in a freezer at − 80 $^{\circ }$C. After the corneal samples were completely frozen, a Cryostatl microtome was used to prepare the frozen specimens. Specimens for every group were then subjected to H &E staining in order to observe cell structure.

#### Epithelial migration

The method used in this experiment was performed as detailed in the report by Harada et al.^6^. First, three corneal blocks (5 $\times$ 3 mm2 each) were prepared from the corneal strips preserved under each of the above-described conditions using a razor blade. Each corneal block was then placed with the epithelial side facing up in the well of a 12-well cell culture plate (CostarTM; Corning Inc., Corning, NY, USA). Subsequently, 1 mL of Ham’s F12 Nutrient Mixture (DMEM/F-12; Thermo Fisher Scientific, Waltham, MA, USA) was added in the 12-well cell culture plate as a basal medium for supporting the growth of corneal epithelial cells. After incubation at 37 $^{\circ }$C for 24 h under humidified conditions of 5% CO$_2$, the corneal blocks were fixed overnight with 4% PFA.

Next, the blocks were immersed in PBS for 1 h and subjected to H &E staining. The stained corneal blocks were attached to the back of the glass surface of a glass-bottomed dish (Matsunami Glass Industries, Ltd., Osaka, Japan) using an Ophthalmic Viscosurgical Device (AMO Japan K.K., Tokyo, Japan).

**Figure 4:**
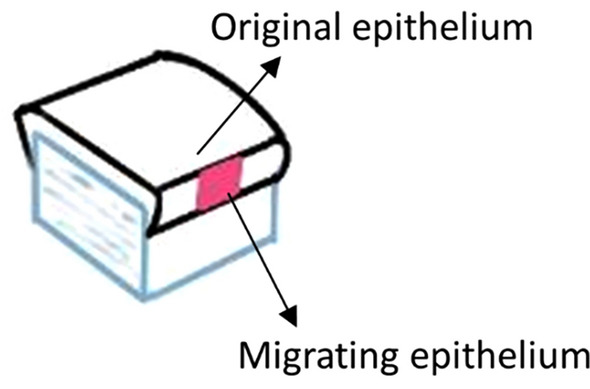
A photograph is taken with a stereomicroscope at two points on the corneal stroma section (longitudinal plane) of each corneal block. The migration area extending to the corneal stroma is then measured 2 mm from the block center, with the two migration areas of each block averaged separately. Image was created with procreate.com.

A photograph was then taken using a stereomicroscope at two points on the corneal stroma section (longitudinal plane) for each of the corneal blocks. The migration area extending to the corneal stroma was subsequently measured 2 mm from the block center (Fig. 4), with the two migration areas of each block averaged separately.

## Results

### Transparency

**Figure 5:**
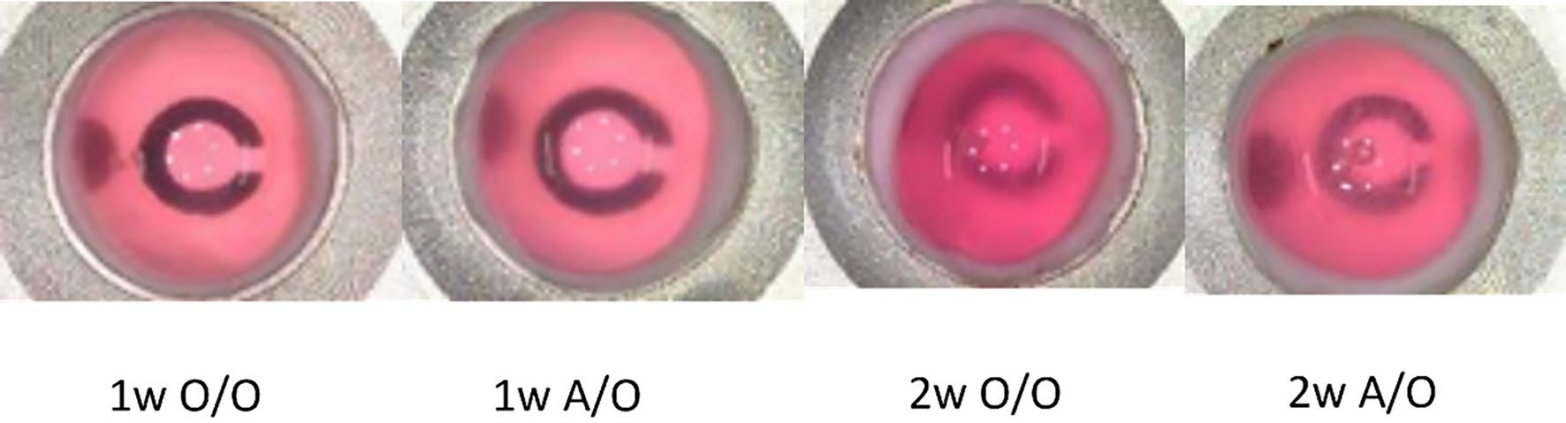
Representative pictures for each group.

The mean scores were 1.3 ± 0.5, 1.7 ± 0.5, 3.2 ± 0.4, 3.2 ± 1.0, respectively, in the 1w O/O, 1w A/O, 2w O/O, and 2w A/O groups. There were no significant differences between the 1w O/O and 1w A/O groups (P = 0.29) or between the 2w O/O and 2w A/O groups (P = 1.0) (Fig. 5).

### Cornea thickness (OCT)

**Figure 6:**
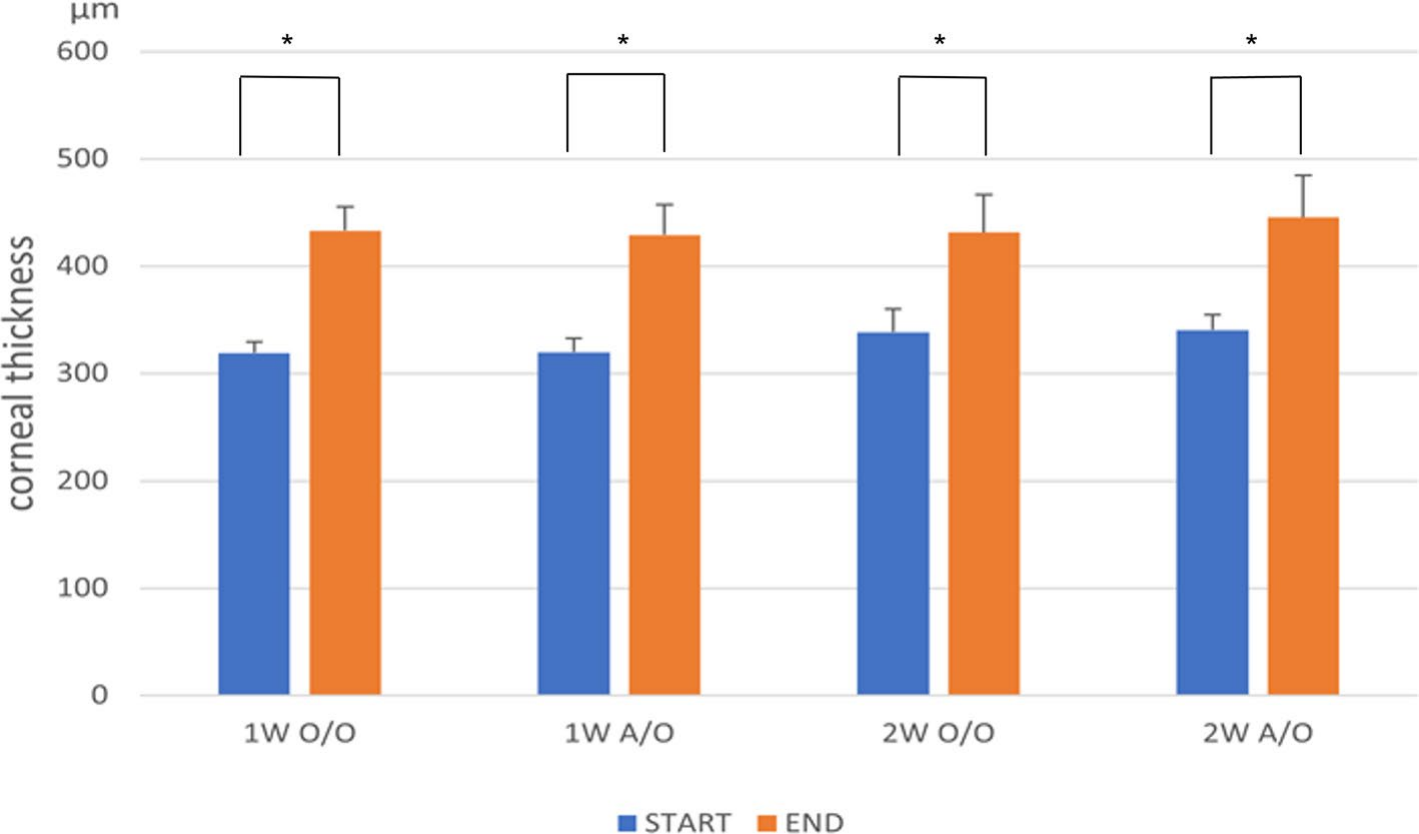
Corneal thickness at the start and end of preservation. For each of the corneas, the thickness at the end is significantly greater than that at the beginning, and there are no significant differences observed among the groups before or after the preservation.

For each cornea, the thickness was significantly greater at the end than at the beginning (P < 0.01) in groups 1w O/O, 1w A/O, 2w O/O, and 2w A/O. However, there were no significant differences observed among the groups before or after preservation (Fig. 6).

### H &E staining

**Figure 7:**
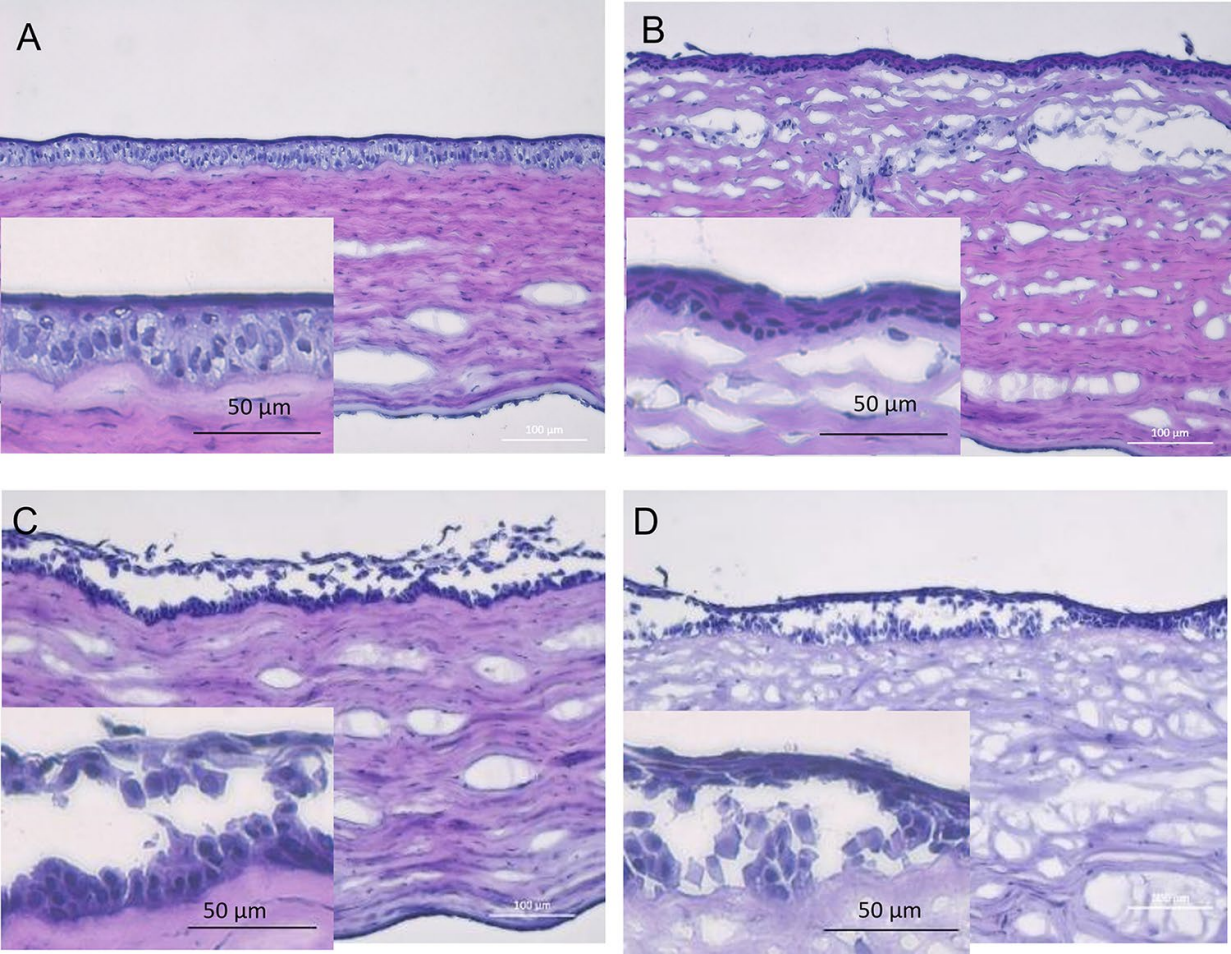
Representative picture for each group: (**A**) 1w O/O, the corneal tissue is structurally intact. (**B**) 1w A/O, the structure of corneal epithelial cells is relatively intact, with no separation observed between the corneal epithelium and stroma. (**C**) 2w O/O, for the separation of the corneal epithelium from the corneal stroma, there is visible separation between the epithelial cell layers or the focal epithelial defects. (**D**) 2w A/O, the structure and morphology of the corneal epithelium can no longer be clearly observed, with the integrity of the corneal epithelium found to be disrupted.

Figure 7 presents the H &E staining results for the four groups of corneas.

For the 1w O/O group, the corneal tissue was structurally intact, and the structure of each layer was clearly visible. The corneal epithelial layer was composed of three or four layers of squamous epithelial cells, which were regularly arranged.

For the 1w A/O group, the structure of the corneal epithelial cells was relatively intact, with no separation observed between the corneal epithelium and stroma. Although the corneal stroma was more edematous compared with the 1w A/O group, the corneal epithelial tissue morphology was basically the same and not significantly different.

For the 2w O/O and 2w A/O groups, after the separation of the corneal epithelium from the corneal stroma, there was a visible separation between epithelial cell layers or focal epithelial defects. The structure and morphology of the corneal epithelium could no longer be clearly observed. In both of these two groups, the integrity of the corneal epithelium was disrupted.

### Epithelial migration

**Figure 8:**
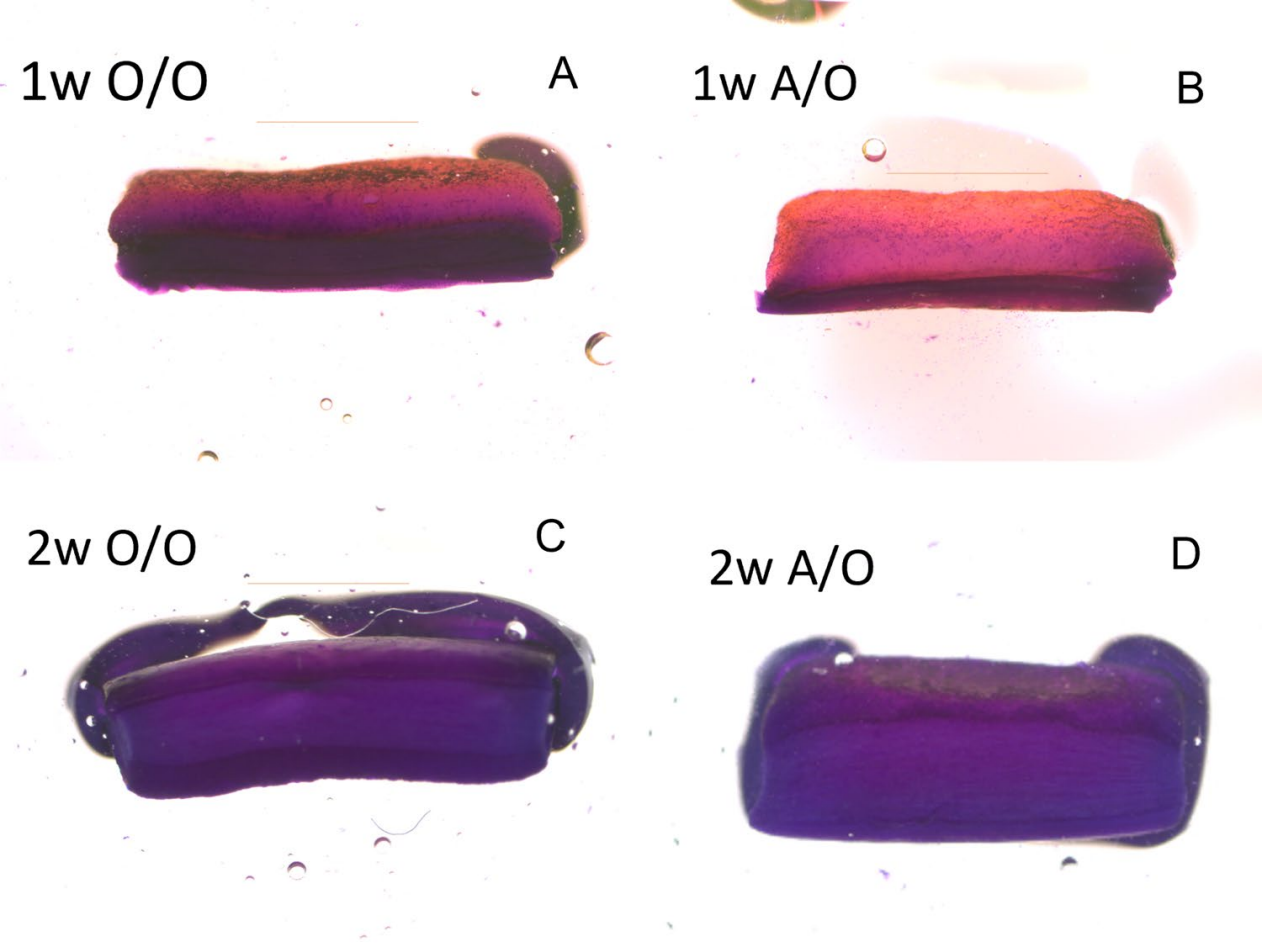
Pictures of corneal epithelial migration. Pictures were taken by ZEN 2.5(blue edition), zeiss.com, images were created with Microsoft PowerPoint,version 2208, microsoft.com. (**A**) 1w O/O, migration from the corneal epithelium from the cut surface to the corneal stroma can be observed. The length from the edge of the corneal epithelial section down to the junction of the corneal epithelium and the corneal stroma is the distance of corneal epithelial migration. Subsequently, the area 2 mm from the block center is measured, making it possible to obtain the area of corneal migration. (**B**) 1w A/O, migration from the corneal epithelium from the cut surface to the corneal stroma is observed. (**C**) 2w O/O, migration of the corneal epithelial cells cannot be observed. (**D**) 2w A/O, migration of the cornea epithelial cells cannot be observed.

Figure 8 presents the pictures of corneal epithelial migration.

**Figure 9:**
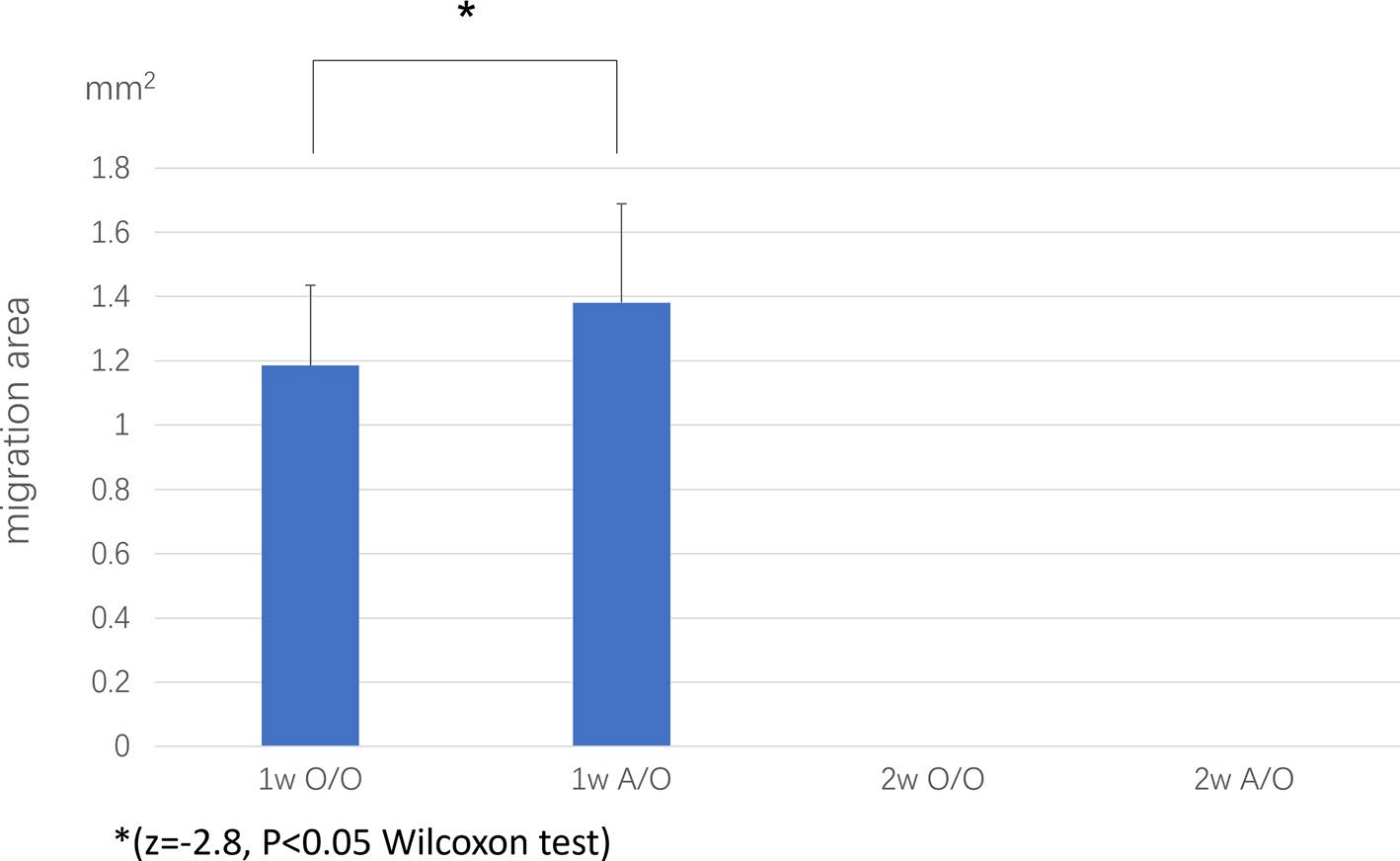
Results of the epithelial migration examinations, comparing the area at the central 2-mm location for each of the samples. Significant differences are observed between the data for the 1-week-preserved Optisol-GS submerged group and the 1-week-preserved airlift group ($\hbox {z}=-2.8, \hbox { p}<0.05$ Wilcoxon test).

Analyses of the central 2-mm area of each of the samples showed that the data for the 1w O/O and 1w A/O groups were significantly different from that shown in Fig. 9 ($\hbox {z}=-2.8, \hbox { p}<0.05$ Wilcoxon test).

There was no epithelial cell migration in the 2w O/O and 2w A/O groups.

## Discussion

In this study, a new method of corneal preservation, in which the endothelium and epithelium of the cornea were preserved separately, was evaluated with four different examinations of the corneas. There was a significant difference in epithelial migration between the tested groups (z = − 2.8, P < 0.05 Wilcoxon test). These results indicate that the corneal epithelium preserved under the one-week airlift conditions showed a larger migration area. However, no differences were found for the other examinations.

The purpose of this study was to evaluate the conditions for a preservation device that was developed for preserving the cornea after separating the epithelial and endothelial areas. This new device allows the epithelium and endothelium of the cornea to be preserved separately. After obtaining a corneoscleral button, it was rinsed with Optisol-GS and then fixed to the single-use anterior chamber. The artificial anterior chamber was then connected to a vertically fixed infusion tube that was filled with Optisol-GS up to 20 cm in height, with pressure maintained at 15 mmHg. This artificial anterior chamber with the button was then placed upside down in a plastic cup, which made it possible to put the samples in different solutions.

In the airlift groups, the corneal endothelium was only immersed in Optisol-GS, while 5 mL of HBSS were placed in the cup, but without any contact with the corneal epithelium. In the Optisol-GS submerged groups, the entire button was completely submerged in the Optisol-GS. The corneal epithelium was preserved using airlift conditions that were comparable to the Optisol-GS conditions.

With regard to the migration of the corneal epithelium, it was shown that the airlift condition was better than the Optisol-GS condition. This suggests a potential advantage for this new preservation method in maintaining the health of corneal epithelial cells. Originally, the airlift method was developed to culture skin cell sheets for transplantation^7,8^. Subsequently, some investigators adopted this method for the culture of corneal epithelial cells. Their study found that the airlift technique reduced the intercellular spaces in the superficial cells and promoted barrier function. This demonstrated the usefulness of the airlift method for culturing corneal epithelial cells for ocular surface reconstruction^9^. Another study showed that preserving corneal limbal tissue by airlift at 4 $^{\circ }$C could help maintain the integrity of the corneal limbal epithelium, as well as maintain the cell-cell junctions^5^. In the present study, the airlift method was used to preserve the corneal epithelium at 4 $^{\circ }$C. It was confirmed that corneal epithelium preserved under airlift conditions for one week maintained the integrity of epithelial cells, and it showed larger corneal epithelial migration.

During corneal transplantation, it is important to maintain the epithelial health of the corneal button. Optisol-GS is a common preservative medium that is used for corneal tissues, and has been shown to be particularly suitable for the preservation of corneal endothelium during hypothermia^10,11^.

However, some reports have suggested that the use of Optisol-GS is not suitable for the preservation of corneal epithelium^11,12^. In contrast, there are other solutions that have been shown to be effective compounds for the preservation of human corneal epithelium^13^.

Following damage to the corneal surface, it is important to accelerate corneal epithelial renewal to avoid transparency and vision loss. Maintaining the integrity of the corneal barrier is also imperative, since this is essential for helping to prevent pathogen penetration^14^. Corneal epithelial wound healing occurs in conjunction with cell proliferation and migration^15^. Cellular reorganization and protein synthesis then occur, with a subsequent loss of the structures responsible for cell adhesion^15^. The cornea then assumes a flattened form and migrates to the wound, thereby covering it^16^. Subsequently, the distal cells then proliferate and migrate to repopulate the wound, which is then followed by cell stratification and differentiation^17^. Finally, there is remodeling of the cell adhesion structures followed by synthesis of the extracellular matrix^18^.

Previous papers that investigated corneal preservation focused primarily on the endothelial condition in the corneal button. After corneal transplantation, the donor’s corneal epithelium will eventually be replaced by the recipient’s epithelial cells. Therefore, more attention has been paid to the importance of the corneal endothelial cells in this corneal preservation process. However, the preservation of corneas lacking corneal epithelium has been reported to promote increased apoptosis of the stromal keratinocytes^12^. Moreover, it has also been reported that the time to corneal epithelial healing after corneal transplantation correlates with the degree of donor corneal epithelial deficiency^19^. This suggests that not only the corneal endothelium, but also the corneal epithelium is important in corneal protection. The procedure of corneal transplantation itself has a risk of damaging the corneal epithelium. While the cornea is punched for preparation of the corneal button, the corneal epithelium may be damaged by direct contact with the punch block. Furthermore, the epithelium may be damaged by forceps, needles, and stiches during suturing of the corneal button. Therefore, corneal epithelium should be maintained as well as possible until the cornea is used for surgery. To reduce the risk of postoperative infection and pain, early recovery of corneal epithelium is important, along with the use of topical antibiotics and lubricants.

Corneal epithelium is a self-renewing tissue with the stem cell niche residing in the corneoscleral junction and limbus, and it provides a life-long supply of proliferating cells for epithelial regeneration^20^. Proper wound healing is dynamic to the maintenance of the integrity and health of the corneal epithelial surface to preserve corneal transparency and vision^20^. Therefore, in limbal allograft transplantation for the treatment of limbal stem cell deficiency (LSCD), the epithelial proliferative potential is especially important in donor corneas^5^. In the current study, the examination of epithelial migration showed that the corneal epithelium preserved under a one-week airlift condition exhibited a larger migration area. If this technique is applied in clinical practice, the 1-week airlift condition could be a new method for improving wound healing after corneal transplantation.

In addition to the corneal epithelial migration experiment, the present study also included three other experiments that were used to verify the status of the corneal epithelial cells, including transparency examination, H &E staining, and corneal thickness tests. The experimental results demonstrated that there were no significant differences in either transparency or corneal thickness between the airlift and Optisol-GS-preserved corneas. These findings suggest that the corneal epithelium preserved under airlift conditions was comparable to that preserved under the Optisol-GS condition.

The current study had two limitations. First, only rabbit corneas were used. Since Japanese law prohibits the use of domestic human donor corneas in academic research, we were not able to examine human corneas. Second, neither immunohistochemistry nor a colony-forming assay was performed to evaluate the proliferative ability of the corneal epithelial cells. However, the migration assay results clearly showed the ability of epithelial migration using the proposed preservation method.

The findings of the current study suggest that corneal epithelium preserved under the hypothermic airlift condition is comparable to that preserved under the Optisol-GS condition, indicating a potential advantage for maintaining the proliferative ability of corneal epithelial cells. Moreover, the present results suggest that there are better conditions for maintaining good corneal health when using this method, such as a better temperature during the airlift condition and a better preservation medium. These factors will need to be further examined in future studies.

## Data Availability

All the relevant data are presented in the manuscript. The datasets generated and analyzed during the current study are available from the corresponding author upon reasonable request.
