# Author Correction: Comparison of rabbit corneal changes during different preservation techniques using optisol-GS and airlift

**DOI:** 10.1038/s41598-023-38917-w

**Published:** 2023-07-19

**Authors:** Diya Tang, Masafumi Uematsu, Kohei Harada, Yasser Helmy Mohamed, Mao Kusano, Daisuke Inoue, Takashi Kitaoka

**Affiliations:** Department of Ophthalmology and Visual Sciences, Graduate School of Biomedical Sciences, Nagasaki University, Nagasaki, 852-8102 Japan

Correction to: *Scientific Reports* 10.1038/s41598-023-34039-5, published online 28 April 2023

The original version of this Article contained an error in Affiliation 1, which was incorrectly given as ‘Ophthalmology, Nagasaki University Hospital, Nagasaki 852-8102, Japan’.

The correct affiliation is listed below:

Department of Ophthalmology and Visual Sciences, Graduate School of Biomedical Sciences, Nagasaki University, Nagasaki, 852-8102, Japan.

The original Article has been corrected.

